# Mass-producible 2D-WS_2_ bulk modified screen printed electrodes towards the hydrogen evolution reaction†

**DOI:** 10.1039/c9ra05342e

**Published:** 2019-08-12

**Authors:** Jack P. Hughes, Felipe D. Blanco, Craig E. Banks, Samuel J. Rowley-Neale

**Affiliations:** Faculty of Science and Engineering, Manchester Metropolitan University Chester Street Manchester M1 5GD UK c.banks@mmu.ac.uk www.craigbanksresearch.com +44(0)1612476831 +44(0)1612471196; Manchester Fuel Cell Innovation Centre, Manchester Metropolitan University Chester Street Manchester M1 5GD UK; University of São Paulo Prof. Lineu Prestes Avenue, Butantã São Paulo 05508-000 SP Brazil

## Abstract

A screen-printable ink that contained varying percentage mass incorporations of two dimensional tungsten disulphide (2D-WS_2_) was produced and utilized to fabricate bespoke printed electrodes (2D-WS_2_-SPEs). These WS_2_-SPEs were then rigorously tested towards the Hydrogen Evolution Reaction (HER) within an acidic media. The mass incorporation of 2D-WS_2_ into the 2D-WS_2_-SPEs was found to critically affect the observed HER catalysis with the larger mass incorporations resulting in more beneficial catalysis. The optimal (largest possible mass of 2D-WS_2_ incorporation) was the 2D-WS_2_-SPE_40%_, which displayed a HER onset potential, Tafel slope value and Turn over Frequency (ToF) of −214 mV (*vs.* RHE), 51.1 mV dec^−1^ and 2.20 
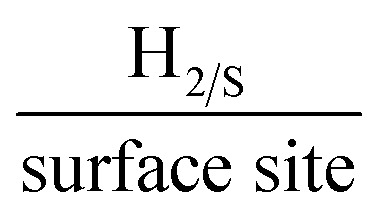
, respectively. These values significantly exceeded the HER catalysis of a bare/unmodified SPE, which had a HER onset and Tafel slope value of −459 mV (*vs.* RHE) and 118 mV dec^−1^, respectively. Clearly, indicating a strong electrocatalytic response from the 2D-WS_2_-SPEs. An investigation of the signal stability of the 2D-WS_2_-SPEs was conducted by performing 1000 repeat cyclic voltammograms (CVs) using a 2D-WS_2_-SPE_10%_ as a representative example. The 2D-WS_2_-SPE_10%_ displayed remarkable stability with no variance in the HER onset potential of *ca.* −268 mV (*vs.* RHE) and a 44.4% increase in the achievable current over the duration of the 1000 CVs. The technique utilized to fabricate these 2D-WS_2_-SPEs can be implemented for a plethora of different materials in order to produce large numbers of uniform and highly reproducible electrodes with bespoke electrochemical signal outputs.

## Abstract 2

Two dimensional tungsten disulphide screen-printed electrodes display efficient electrocatalysis towards the hydrogen evolution reaction.

## Graphical abstract

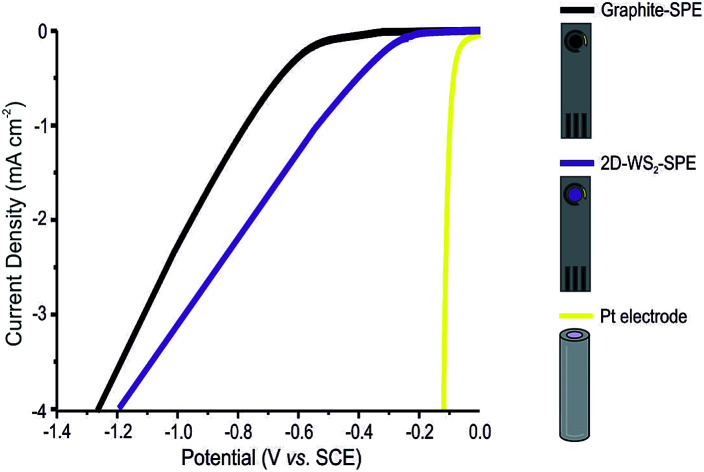

## 1. Introduction

The global reliance on fossil fuels has incited scientists and governing bodies to make the transition from unsustainable to clean energy solutions, such as recent developments in renewable hydrogen energy.^1–3^ The potential to implement hydrogen as a less polluting alternative to fossil fuel based energy generation and storage techniques, has become more economically feasible as alternatives to the precious metal catalysts, required for efficient water splitting, have been developed.^4–6^ Electrolysers are commonly used to produce ‘green’ hydrogen *via* water splitting with the energy requirement being met by renewable sources, *e.g.* wind, wave and solar.^7–9^ The Hydrogen Evolution Reaction (HER) and the Oxygen Evolution Reaction (OER) are the major reactions occurring within an electrolyser.^10,11^ The efficiency of the HER is dependent on the choice of electrocatalyst, with Pt being considered the optimal electrocatalyst currently utilized within commercial electrolysers.^12–15^ An interesting study by Weng *et al.*^16^ utilised AuPt alloy nanodendrites (AuPt NDs) anchored upon a glassy carbon electrode (GCE), where excellent HER catalysis was exhibited with an overpotential and Tafel value of −27 mV (*vs.* RHE) and 34 mV dec^−1^, respectively. However, large-scale usage of precious metals is limited by high cost and relatively low earthly abundance,^17–19^ therefore research has been focused on developing electrocatalysts that are cheaper, more earth abundant and can be implemented with significant scalability. One such HER catalyst was developed by Wang *et al.*,^20^ whom modified a GCE with an N-doped graphene tungsten carbide composite (W_*x*_C/NG-10), which improved the overpotential from −484 mV (*vs.* RHE) to −150 mV (*vs.* RHE).

Certain transition metal dichalcogenides (TMD's), such as 2D-MoS_2_ and 2D-WS_2_, have been shown to display promising HER catalysis.^21–25^ Yao *et al.*^26^ doped a GCE with 2D-WS_2_, anchored upon a graphdiyne framework (GD-WS_2_), where the HER onset potential was improved from −350 mV to −140 mV, regarding bare graphdiyne to the modified GD-WS_2_. At the atomic level, a 2D-WS_2_ nanosheet is a single tier of tungsten metal atoms enclosed by two layers of sulphur atoms, with weak van der Waals forces binding each layer.^27^ Catalytic active sites are observed to be located at the edge sites of a 2D-WS_2_ monolayer. In comparison with the (0001) basal planes of 2D-WS_2_, the edge planes possess distinct physical, electronic and stoichiometric properties. An increased number of active edge states within the structural design of TMD's corresponds to enhanced HER kinetics.^28^ Density functional theory (DFT) binding energy calculations demonstrate that the suspended bonds of S atoms are electronegatively charged and have an affinity for H^+^ atoms within acidic media,^29^ represented by a binding energy of 0.06 eV.^30^Table 1 exhibits a thorough overview of the current literature studies that utilise tungsten based electrocatalytic materials towards the HER. The use of 0.5 M H_2_SO_4_ as an electrolyte is common practice whilst exploring a material towards the HER, where the conductive abilities of acidic electrolyte solutions in comparison to alkaline solutions are believed to allow the HER to proceed at a faster rate.^31^ Although the studies in Table 1 are competent in their approach to utilizing tungsten-based electrocatalysts towards the HER, there is a continual application of glassy carbon as a supporting electrode for the deposition of the catalyst material. Neither GC or Pt are scalable in fuel cell technologies due to their high cost, this also applies to supporting materials utilised in other studies in Table 1 such as Au. The screen printing and exfoliation of electroactive nanomaterial upon the surface of an electrode allows for uniform dispersion of catalyst coupled with the reduced requirement of solvent within the catalyst mixture, where resultant electrodes are suitable for industrial application.^32,33^ The utilization of screen-printed electrodes (SPEs) as supporting materials is an attractive alternative due to their significantly lower expense, scalable reproducibility and the ordered precision with which the working area can be fabricated.

**Table 1:** Comparison of current literature using WS_2_ and related catalytic materials towards the HER^a^

Catalyst	Supporting electrode	Electrolyte	Deposition technique	HER/OER onset potential	Tafel value	Ref.
WS_2_	NPG	0.5 M H_2_SO_4_	Electrochemical reduction	−0.11 V (*vs.* RHE)	74 mV dec^−1^	55
W_*x*_C/NG	GC	0.5 M H_2_SO_4_	Drop casting	−0.078 V (*vs.* RHE)	45.9 mV dec^−1^	20
WC nanowall	SW	0.5 M H_2_SO_4_	Plasma assisted deposition	−0.052 V (*vs.* RHE)	67 mV dec^−1^	56
WS_2_	GC	0.5 M H_2_SO_4_	Drop casting	−0.10 V (*vs.* RHE)	48 mV dec^−1^	49
WSe_2_P	CF	0.5 M H_2_SO_4_	High temperature/drop casting	−0.22 V (*vs.* RHE)	69 mV dec^−1^	57
WC nanotubes	GC	0.5 M H_2_SO_4_	Drop casting	−0.31 V (*vs.* RHE)	69 mV dec^−1^	58
rGO/WS_2_/WO_3_	GC	0.5 M H_2_SO_4_	Drop casting	−0.09 V (*vs.* RHE)	37 mV dec^−1^	59
N-WC	CFP	0.5 M H_2_SO_4_	Drop casting	−0.30 V (*vs.* RHE)	149 mV dec^−1^	60
W_2_C	GC	0.5 M H_2_SO_4_	Spin coating	−0.30 V (*vs.* RHE)	145 mV dec^−1^	61
WS_2_/W	GC	0.5 M H_2_SO_4_	Drop casting	−0.14 V (*vs.* RHE)	46.8 mV dec^−1^	50
W_2_C/WP	GC	0.5 M H_2_SO_4_	Drop casting	−0.10 V (*vs.* RHE)	61 mV dec^−1^	62
Fe-WO_*x*_P/rGO	GC	0.5 M H_2_SO_4_	Drop casting	−0.075 V (*vs.* RHE)	41.9 mV dec^−1^	63
W_2_C-NC-WN	GC	0.5 M H_2_SO_4_	Drop casting	−0.13 V (*vs.* RHE)	96.4 mV dec^−1^	64
2D-WS_2_-SPE_5%_	SPE	0.5 M H_2_SO_4_	Screen printing	−0.28 V (*vs.* RHE)	111 mV dec^−1^	This work
2D-WS_2_-SPE_10%_	SPE	0.5 M H_2_SO_4_	Screen printing	−0.27 V (*vs.* RHE)	70.5 mV dec^−1^	This work
2D-WS_2_-SPE_20%_	SPE	0.5 M H_2_SO_4_	Screen printing	−0.23 V (*vs.* RHE)	64.9 mV dec^−1^	This work
2D-WS_2_-SPE_40%_	SPE	0.5 M H_2_SO_4_	Screen printing	−0.21 V (*vs.* RHE)	51.1 mV dec^−1^	This work

a NPG; nanoporous gold, GC; glassy carbon, SW; silicon wafer, CF; copper foil, CFP; carbon filter paper, WS_2_; tungsten disulfide, WC; tungsten carbide, WSe_2_; tungsten diselenide, P; phosphide, rGO; reduced graphene oxide, WO_3_; tungsten trioxide, NC; nitrogen carbide, WN; tungsten nitride, Mo; molybdenum, RHE; reversible hydrogen electrode, SPE; screen printed electrode.

The increasing scarcity of fossil fuels has prompted the implementation of hydrogen energy as an alternative means of energy production and storage. Within this study we produce SPEs with WS_2_ incorporated into their bulk structure that exhibit excellent HER catalysis. The issue of poor stability that can arise when exploring a material by drop-casting it onto the surface of a substrate electrode is eliminated by anchoring the WS_2_ into the graphitic ink used in SPEs.^34,35^ We have produced cost effective and highly reproducible 2D-WS_2_-SPEs that may be viewed as alternatives to Pt based materials that are implemented as cathodic materials within modern electrolysers.

## 2. Experimental section

### 2.1 Chemicals

All chemicals used were of analytical grade from Sigma Aldrich and were without need of any further purification. The WS_2_ nanopowder (product code: 790583; Sigma Aldrich, U.K.)^36^ utilised was of 99% purity (trace metals basis, 90 nm avg. part size (SEM)). Electrochemical measurements were performed in 0.5 M H_2_SO_4_, which was of the highest possible purity from Sigma Aldrich (99.999%, double distilled for trace metal analysis).

### 2.2 Electrochemical measurements

The electrolyte (0.5 M H_2_SO_4_) was made using deionized water (resistivity 18.2 MΩ cm^−2^), which was degassed with high purity nitrogen preceding electrochemical measurements. It is prevalent within research conducting HER experiments to remove any trace of oxygen, to prevent the onset of the competing OER.^20,37^ An Autolab Compact™ (Switzerland) potentiostat was used to carry out electrochemical measurements. A three-electrode system was used to take measurements with graphitic screen printed electrodes (SPEs) with a working area diameter of 3.1 mm used as working electrodes, with a carbon counter and a saturated calomel electrode (SCE) reference. The screen printing process used to produce the 2D-WS_2_-SPEs utilised within this study is explained within the ESI† and is also described in more depth in previous studies.^34,35,38,39^ Herein, the potential is referenced to the reversible hydrogen electrode (RHE) utilising the Nernst equation;^40^

1 *E*_RHE_ = *E*_SCE_ + 0.059pH + 0.2415 V (at 25 °C)

## 3. Results and discussion

### 3.1 Characterisation of the 2D-WS_2_ utilised in the fabrication of the 2D-WS_2_-SPEs

The equipment specifications used for the characterisation of the commercially sourced 2D-WS_2_ powder utilised in the fabrication of the 2D-WS_2_-SPEs is described within the ESI.†

**Fig. 1:**
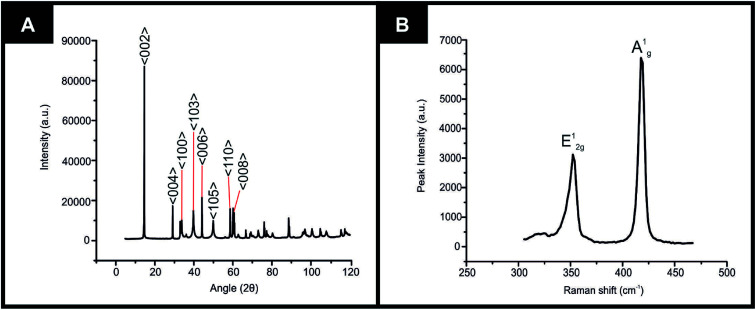
Characterization of commercially sourced 2D-WS_2_ (A) XRD spectra of the 2D-WS_2_, (B) Raman spectra of the 2D-WS_2_.

**Fig. 2:**
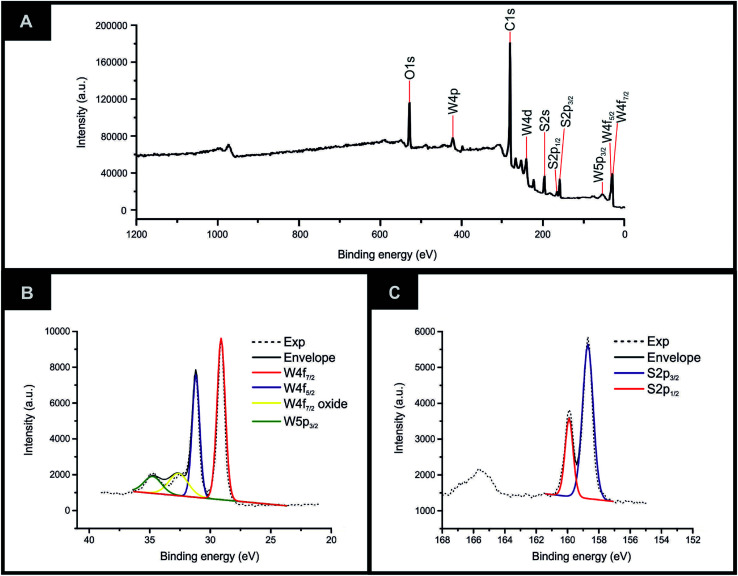
Characterization of commercially sourced 2D-WS_2_ (A) high resolution XPS survey spectrum of the 2D-WS_2_ (wide fit), (B and C) high resolution XPS spectra of the W5p, W4f and S2p regions in 2D-WS_2_, respectively.

XRD could be utilised to assess the crystal structure of the 2D-WS_2_ powder. XRD analysis of the 2D-WS_2_ is presented in Fig. 1(A), where characteristic diffraction peaks at 2*θ* = 14.3°, 29.0°, 32.9°, 39.6°, 44.2°, 49.9°, 58.6° and 60.0° are indexed towards the following pure hexagonal WS_2_ faces, respectively; (002), (004), (100), (103), (006), (105), (110) and (008).^41^ After XRD analysis exhibited the crystal structure of 2D-WS_2_, Raman spectroscopy was used to further confirm the purity of the 2D-WS_2_ powder. Raman spectra displayed in Fig. 1(B), exhibited characteristic wavenumbers at *ca.* 337 and 413 cm^−1^ which correspond to E^1^_2g_ and A^1^_g_ vibrational bands, respectively.^42,43^ The E^1^_2g_ signal conforms with the in plane mode, whereas the signal for the A^1^_g_ vibrational band denotes the out of plane mode.^44^ Next, elemental analysis was carried out using XPS. Fig. 2(A) depicts the high resolution XPS survey spectrum of the 2D-WS_2_ (wide fit). Fig. 2(B) highlights the presence of the binding energies for W5p^3/2^, W4f^5/2^ and W4f^7/2^ at 37.5, 34.0 and 31.5 eV, repectively, in which the W5p^3/2^ peak corresponds to the W^6+^ species and the W^4+^ oxidation state is represented by the peaks for W4f^5/2^ and W4f^7/2^.^45,46^ Shown in Fig. 2(C) are the binding energies for S2p^1/2^ and S2p^3/2^ at 167.5 and 166.2 eV, ascribed to S^2−^ in WS_2_.^47,48^ It is evident from the above physicochemical analysis of the commercially sourced 2D-WS_2_ powder used to formulate the electrocatalytic inks, that the 2D-WS_2_ used within this study is composed of high quality 2D-nanosheets. It was therefore utilised to produce SPEs, which contained a 5, 10, 20 and 40% mass incorporation. These produced SPEs were denoted as 2D-WS_2_-SPE_5%_, 2D-WS_2_-SPE_10%_, 2D-WS_2_-SPE_20%_ and 2D-WS_2_-SPE_40%_.

**Fig. 3:**
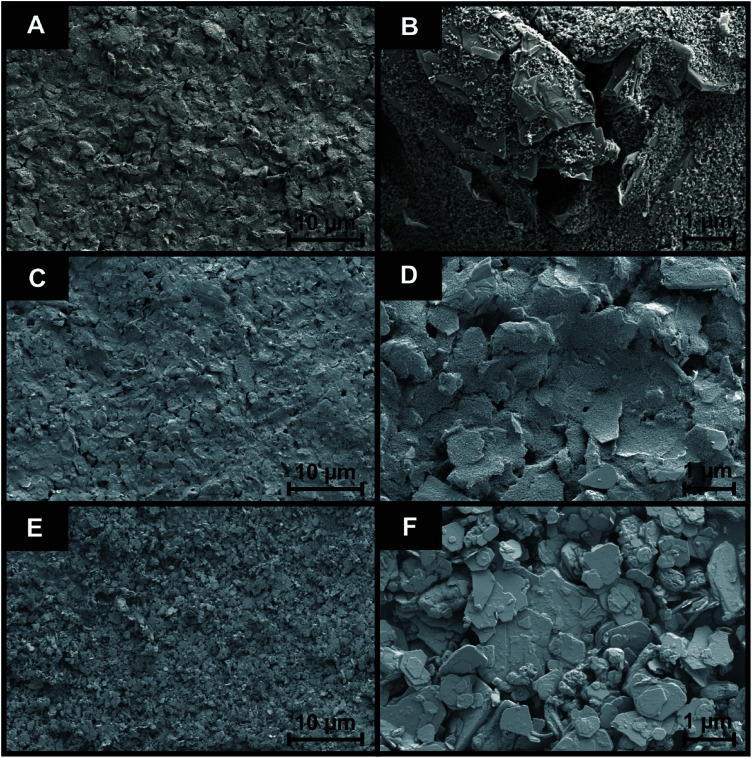
SEM images of the surface of a bare/unmodified SPE (A and B) and the 2D-WS_2_-SPE_10%_ (C and D). (E and F) represent images of the WS_2_ powder. SEM magnifications of ×1k (scale bar, 10 μm) and ×10k (scale bar, 1 μm) for the images (A to B, C to D and E to F) were used, respectively.

Following the fabrication of the 2D-WS_2_-SPEs it was important to assess the surface coverage of 2D-WS_2_ upon the SPE. SEM analysis displayed in Fig. 3 presents SEM images of the surface of a bare/unmodified SPE (A and B) and the 2D-WS_2_-SPE_10%_ (C and D), whilst (E and F) represent images of the 2D-WS_2_ powder. Fig. 3(E and F) supports the 90 nm average particle size stated by the commercial supplier of the 2D-WS_2_ powder.^36^ SEM analysis provides unsubstantial evidence for complete uniform coverage under visual assessment, as the modified WS_2_ surface could not be distinguished from the underlying SPE layer. Raman mapping of the electrode surface, presented in Fig. S1,† was employed to assess whether screen printing led to uniform coverage of WS_2_ on the graphitic surface of the 2D-WS_2_-SPE_5%_, 2D-WS_2_-SPE_10%_, 2D-WS_2_-SPE_20%_, and 2D-WS_2_-SPE_40%_ (Fig. S1(A, B, C and D)†, respectively). When comparing the underlying graphite peak area at *ca.* 1580 cm^−1^ against the area of the WS_2_ Raman peaks at *ca.* 351 cm^−1^, the surface area coverage of the electrode could be investigated. Clearly increasing the mass deposition of WS_2_ on the SPE surface corresponds to an increased Raman intensity in the respective assigned peaks. Analysis of the Raman maps depicted in Fig. S1(B–D)† suggest complete coverage of 2D-WS_2_ upon the underlying SPE support material (which has a surface area of 0.0707 cm^2^), for the electrodes 2D-WS_2_-SPE_10%_, 2D-WS_2_-SPE_20%_, and 2D-WS_2_-SPE_40%_.

### 3.2 Application of the 2D-WS_2_-SPEs towards the HER

**Fig. 4:**
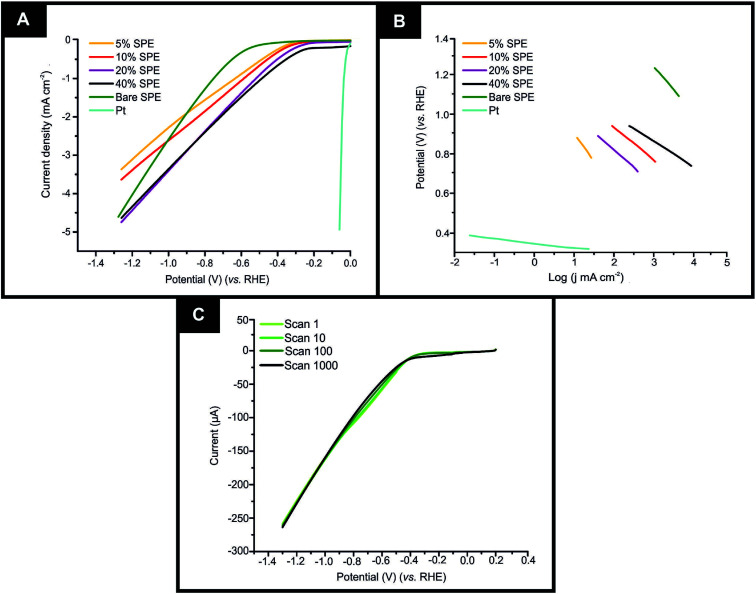
(A) Linear sweep voltammetry (LSV) of a bare/unmodified SPE and the 2D-WS_2_-SPE_5%_, 2D-WS_2_-SPE_10%_, 2D-WS_2_-SPE_20%_, and 2D-WS_2_-SPE_40%_, exhibiting the onset potential of the HER, in the potential range 0.2 to −1.2 V (*vs.* SCE). Scan rate: 25 mV s^−1^ (*vs.* RHE) in 0.5 M H_2_SO_4_. (B) Tafel analysis; potential (*vs.* RHE) *vs.* log(current density) (*j* mA cm^−2^) for the faradaic region of the LSV displayed in (A). (C) Cycling stability testing of the 2D-WS_2_-SPE_10%_*via* LSV in the potential window 0.2 to −1.2 V (*vs.* SCE) at a scan rate of 100 mV s^−1^ (*vs.* RHE), repeated for 1000 cycles.

It is important to initially benchmark the electrocatalytic performance of 2D-WS_2_-SPEs towards the HER against a bare/unmodified SPE and the optimal polycrystalline Pt electrode in 0.5 M H_2_SO_4_. The HER onset is determined as the potential at which the observed current deviates from the background current by the value of 25 μA cm^−2^.^49^ Linear sweep voltammetry (LSV) is exhibited in Fig. 4(A), outlining the electrochemical performance of a bare/unmodified SPE, a polycrystalline Pt electrode and the 2D-WS_2_-SPE_5%_, 2D-WS_2_-SPE_10%_, 2D-WS_2_-SPE_20%_, and 2D-WS_2_-SPE_40%_. The bare/unmodified SPE and Pt electrode exhibited HER onset potentials of −459 mV (*vs.* RHE) and −0.03 V (*vs.* RHE), respectively. The ability of Pt as an electrocatalytic material is explained by its small binding affinity for H^+^ ions. The 2D-WS_2_-SPE_5%_, 2D-WS_2_-SPE_10%_, 2D-WS_2_-SPE_20%_ and 2D-WS_2_-SPE_40%_ were then explored towards the HER and found to exhibit HER onset values of −0.28, −0.27, −0.23 and −0.21 V (*vs.* RHE), respectively. All of the 2D-WS_2_-SPEs exhibit a less electronegative and therefore more favourable HER onset potential compared to that of a bare/unmodified SPE. It is clear that higher percentage incorporation of WS_2_ within the SPEs results in a lower HER onset value, this is likely a result of the increase in the number of electrocatalytic active 2D-WS_2_ edge sites on the SPEs surface. There was a corresponding increase in the achievable current density of the modified SPEs as the percentage mass incorporation of 2D-WS_2_ increases.

The decrease in electronegativity of the HER onset potential coupled with the increase in observed current density across the range of 2D-WS_2_-SPEs suggests there was an alteration in the reaction mechanism. In order to determine whether the increased HER performance arises from the alteration in reaction mechanism, Tafel analysis was performed.^50^ There are three possible courses by which the reaction may progress, where each may be the rate determining step. The first step is the Volmer reaction, which infers the initial adsorption of H^+^, characterised by a Tafel slope value of 120 mV s^−1^. The Volmer step may then be followed by the Heyrovsky or the Tafel discharge steps, which are characterised by Tafel slope values of 40 and 30 mV s^−1^, respectively.^51–53^Fig. 4(B) displays the Tafel analysis extrapolated from the faradaic regions of the LSV's presented in Fig. 4(A). Tafel values of 118, 111, 71, 65 and 51 mV dec^−1^ correspond to a bare/unmodified SPE and the 2D-WS_2_-SPE_5%_, 2D-WS_2_-SPE_10%_, 2D-WS_2_-SPE_20%_, and 2D-WS_2_-SPE_40%_, respectively. The rate limiting step of the HER reaction mechanism for a bare/unmodified SPE is the Volmer step, where it is also suggested by analysis of Tafel values that the rate limiting step for the 2D-WS_2_-SPE_5%_, is also the Volmer step. However, it is observed that the 2D-WS_2_-SPE_10%_, 2D-WS_2_-SPE_20%_ and 2D-WS_2_-SPE_40%_ possess a rate limiting step described by the more beneficial discharge Herovsky step.^54^ In regards to HER onset, achievable current density and the reaction mechanism, the 2D-WS_2_-SPE_40%_ displays the optimal activity. This is likely due to the 2D-WS_2_-SPE_40%_ having the largest number of electrocatalytically active edge sites available upon the SPEs surface (as suggested by the Raman mapping within Fig. S1†) and therefore the highest affinity for binding with H^+^. In order to deduce the effect that increasing the mass incorporation of WS_2_ had upon the intrinsic electrocatalysis exhibited by the 2D-WS_2_ on a per active site basis, a H_2_ Turn over Frequency (ToF) calculation was performed on the modified SPEs. These ToF calculations are presented in the ESI.† The 2D-WS_2_-SPE_5%_, 2D-WS_2_-SPE_10%_, 2D-WS_2_-SPE_20%_ and 2D-WS_2_-SPE_40%_ yielded ToF values of 0.31, 1.20, 1.33 and 2.20 
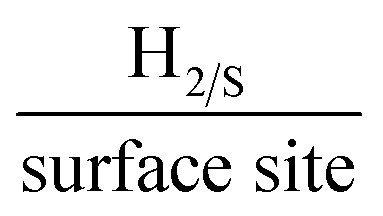
, respectively. The ToF values obtained for each 2D-WS_2_-SPE are again in agreement with the Raman mapping trend presented in Fig. S1,† where increased uniform coverage on the surface of the electrode leads to enhanced electrochemical performance towards the HER, in terms of HER onset potential and Tafel values and also the frequency at which hydrogen is turned over per active site basis.

If the electrochemical technology employed within this study is to be applicable for industrial scale usage, the stability, and therefore the longevity of the electrodes electrocatalytic functionality is to be assessed. Therefore a cycling stability test was carried out in the potential range 0 to −1.5 V, using the 2D-WS_2_-SPE_10%_ where a 44.4% increase in the current at the HER onset potential from 13.4 to 24.1 uA corresponding to the 1^st^ scan to the 1000^th^ scan was observed (shown in Fig. 4(C)). Analysis of Fig. 4(C) indicates that there is no change in the HER onset potential at *ca.* −268 mV (*vs.* RHE) from the 1^st^ to the 1000^th^ scan, hence inferring no depletion of electrocatalytic functionality. The increase in current observed throughout the cycling stability test is likely a result of sustained exposure of the WS_2_ electrocatalytic surface to the 0.5 M H_2_SO_4_ electrolyte, resulting in the stripping of a minimal number of WS_2_ nanosheets to yield an electrocatalytic surface with a greater number of exposed active sites.

**Fig. 5:**
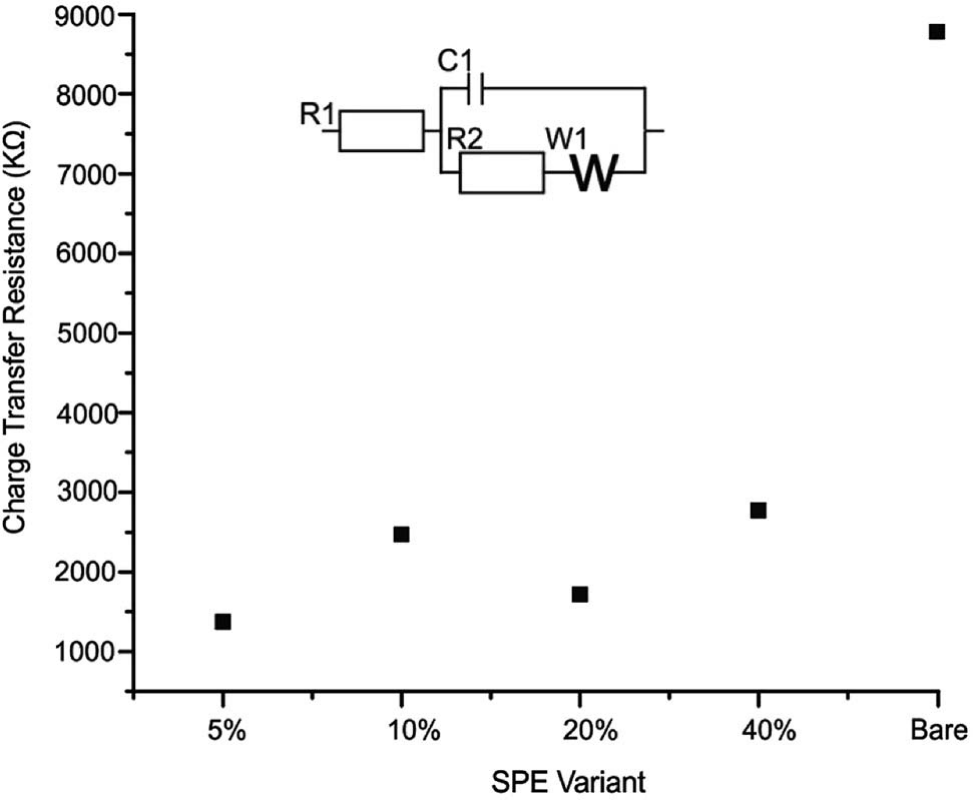
Electrochemical impedance spectroscopy (EIS) displaying the charge transfer resistance of each SPE variant utilised in this study; 2D-WS_2_-SPE_5%_, 2D-WS_2_-SPE_10%_, 2D-WS_2_-SPE_20%_, 2D-WS_2_-SPE_40%_ and bare/unmodified. The frequency range was from 0.1–100 000 Hz at an amplitude of 10 mV (*vs.* RHE) using 1 mM potassium ferrocyanide (II)/ferricyanide (III) in 0.1 M KCl. Inset shows equivalent circuit.

The effective resistance or impedance of the electrical circuit was measured for a bare/unmodified SPE, 2D-WS_2_-SPE_5%_, 2D-WS_2_-SPE_10%_, 2D-WS_2_-SPE_20%_ and 2D-WS_2_-SPE_40%_. Electrical impendance spectroscopy (EIS), at the frequency range 0.1–100 000 Hz and amplitude of 10 mV (*vs.* RHE) using 1 mM potassium ferrocyanide (II)/ferricyanide (III) in 0.1 M KCl was used to plot each 2D-WS_2_-SPE variant against the charge transfer resistance (*Ω*) observed for each electrode (see Fig. 5). All 2D-WS_2_-SPEs exhibit a significantly reduced charge transfer resistance comparable to a bare/unmodified SPE, which has a *Ω* value of 8.8 × 10^3^*Ω*. Charge transfer resistance values of 1.4 × 10^3^, 2.5 × 10^3^, 1.7 × 10^3^ and 2.8 × 10^3^ Ω, coincide with the electrodes 2D-WS_2_-SPE_5%_, 2D-WS_2_-SPE_10%_, 2D-WS_2_-SPE_20%_ and 2D-WS_2_-SPE_40%,_ respectively. It is evident that the 2D-WS_2_-SPE_40%_ is responsible for the largest *Ω* value, where bulk coagulation of WS_2_ at the electrode surface is likely responsible for the realized increase in resistance.

A percentage relative standard deviation (% RSD) calculation was carried out to assess the intrarepeatability of the 2D-WS_2_-SPEs (*N* = 3). RSD values of 0.11, 0.34, 0.56 and 0.97%, represent the HER onset potential deviation for the 2D-WS_2_-SPE_5%_, 2D-WS_2_-SPE_10%_, 2D-WS_2_-SPE_20%_ and 2D-WS_2_-SPE_40%_, respectively. Concerning the RSD of current density at −0.51 V (*vs.* RHE) there is no overall trend, whereas there is an observable increasing trend with respect to the RSD of the HER onset potential of each 2D-WS_2_-SPE as percentage mass loading increases. We hypothesize that the increasing trend in RSD referring to the HER onset potential of the 2D-WS_2_-SPEs is a result of the increased deposition of WS_2_, leading to the presence of a larger edge to basal plane ratio, hence more available active sites providing increased variation within the topology of each electrode. Moreover, the % RSD values reported within this study indicate reproducibility of the screen printing technique utilized to deposit WS_2_ upon the working area of an SPE.

The various advantages of SPEs comparable to conventional carbon based electrodes are apparent by the means of their application to wide scale industrial usage, nevertheless, electrochemical activity towards the HER remains a factor where vast improvements could be made and implemented. We address this topic herein by fabricating SPEs with 2D-WS_2_ bulk modified inks that significantly reduce the HER onset potential whilst increasing the achievable current density. The incorporation of these inks onto an SPE surface exhibit a desirable cycling stability when compared to the universally implemented technique of drop casting. Although the WS_2_-SPEs are observed at a more electronegative HER onset potential than Pt, they infer low cost and excellent reproducibility over a short timescale. These factors are indicative that 2D-WS_2_-SPEs may be seen as a viable alternative to the Pt electrode used throughout literature.

## 4. Conclusions

We have incorporated 2D-WS_2_ into the bulk of screen printed electrodes, by means of % mass incorporation. Raman mapping demonstrated complete uniform coverage of 2D-WS_2_ upon the surface of the SPEs after 10% mass incorporation, where a higher % mass incorporation of 2D-WS_2_ within the SPEs enhanced the electrochemical activity towards the HER. The 2D-WS_2_-SPE_40%_ exhibits optimal electrocatalytic ability with regards to HER onset potential, Tafel value and turnover frequency (ToF) with values of −214 mV (*vs.* RHE), 51.1 mV dec^−1^ and 2.20 
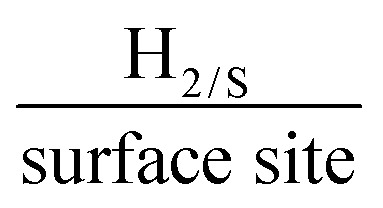
, respectively. The electrocatalytic activity of a bare SPE towards the HER is −459 mV (*vs.* RHE) and 118 mV dec^−1^ in terms of the HER onset potential and Tafel value, respectively, therefore we can state that the 2D-WS_2_-SPEs utilised herein exhibit excellent electrocatalytic ability. Cycling stability measurements of the 2D-WS_2_-SPE_10%_ showed there was no observed degradation in HER onset potential over 1000 repeat scans and a marked increase in current of 44.4% was observed. The mechanism by which the HER proceeds and the significance of mass ratios in the WS_2_ electrocatalytic inks has been presented. The highly reproducible electrode production procedure may be utilised in future research at a cheap cost. For the first time, 2D-WS_2_-SPEs have been produced and their electrochemical performance has been explored towards the HER. Their potential to be utilised as the cathodic material within electrolysers has been displayed.

## Conflicts of interest

There are no conflicts to declare.

## Supplementary Material

RA-009-C9RA05342E-s001
