# Supplementary material for: Mass-producible 2D-WS2 bulk modified screen printed electrodes towards the hydrogen evolution reaction
Source: RSC Adv. 2019 Aug 12;9(43):25003–11. doi: 10.1039/c9ra05342e (PMC9069938; doi:10.1039/c9ra05342e)

Electronic Supplementary Material (ESI) for RSC Advances.
This journal is © The Royal Society of Chemistry 2019

Electronic Supporting Information (ESI) for the following publication:

Mass-Producible 2D-WS_2 Bulk Modified Screen Printed Electrodes towards the Hydrogen Evolution Reaction

Jack P. Hughes[1,2], Felipe D. Blanco[3], Craig E. Banks[1,2*] and Samuel J. Rowley-Neale[1,2*]

1 : Faculty of Science and Engineering, Manchester Metropolitan University, Chester Street, Manchester M1 5GD, UK.

[2]: Manchester Fuel Cell Innovation Centre, Manchester Metropolitan University, Chester Street, Manchester M1 5GD, UK.

[3]: University of São Paulo, Prof. Lineu Prestes Avenue, Butantã, São Paulo 05508-000, SP, Brazil.

*To whom correspondence should be addressed.

Email: c.banks@mmu.ac.uk; Tel: ++(0)1612471196; Fax: ++(0)1612476831

Website: www.craigbanksresearch.com

2D-WS_2-SPE Production

The working electrodes were incorporated with 2D-WS_2 internally using specialised stencil screens within the DEK 248 screen-printing unit. (DEK, Weymouth, UK). The incorporation of the 2D-WS_2 electrocatalytic inks started with the printing of a carbon-graphite ink (product code: C2000802P2; Gwent Electronic Materials Ltd., U.K.) layer onto a polyester (Autostat, 250 μm thickness) substrate. The layer was then cured at 60ºC for 30 minutes in a fan oven. The connections were sealed with a dielectric paste (product code: D2070423D5; Gwent Electronic Materials Ltd., U.K.) and the electrodes were ready to use after curing at 60ºC for 30 minutes.

Incorporation of the 2D-WS_2 powder into a carbon-graphitic ink was carried out using weight percentage of M_P to M_I, where M_P is the mass of particulate (the mass of WS_2) and M_I is the total mass of the ink including the base graphitic ink and the mass of the particulate. Therefore the equation $(M_P/M_I)\times100$ was used to formulate four ink compositions for WS_2 in the weight percentage range 5, 10, 20 and 40%.

Characterisation Equipment

Scanning electron microscope (SEM) images were obtained using a JEOL JSM-5600LV model SEM equipped with an energy-dispersive X-ray microanalysis (EDS) package.

X-ray powder diffraction (XRD) data was collected using a PANalytical X'Pert diffractometer fitted with a PixCEL 1-D detector using a Cu anode ($k_{\alpha}1$ λ= 1.5406Å) with the generator set at 40 mA, 40 kV. Data was collected in the range 5-120° 2θ with a step size of 0.013° 2θ and a collection time of 118 s step^{-1} using automatic divergence and antiscatter slits with an observed length of 5.0 mm. Data was processed using HighScore Plus version 4.7 (PANalytical BV, Delft, Netherlands, 2017). Raman spectroscopy was performed using a 'Renishaw InVia' spectrometer equipped with a confocal microscope (×50 objective) and an argon laser (514.3 nm excitation). Heating effects were avoided by performing measuremnts at a very low laser power level (0.8 mW).

The X-ray photoelectron spectroscopy (XPS) data was acquired utilsing a bespoke ultra-high vacuum system equipped with a Specs GmbH Focus 500 monochromated Al Kα X-ray source, Specs GmbH Phoibos 150 mm mean radius hemispherical analyser with 9-channeltron detection, and a Specs GmbH FG20 charge neutralising electron gun. Survey spectra were obtained over the binding energy range 1200–0 eV using a pass energy of 50 eV and high resolution scans were made over the C 1s and O 1s lines using a pass energy of 20 eV. The analysis used the mean area over a region approximately 1.4 mm in diameter on the sample surface, using the 7 mm diameter aperture and lens magnification of ×5. The energy scale of the instrument is calibrated according to ISO 15472, and the intensity scale is calibrated using an in-house method traceable to the UK National Physical Laboratory.[65] Data interpretation was carried out using CasaXPS software v2.3.16.4.

Turn over frequency calculation (ToF)

Evaluation of how varying percentage of ink modification alters the catalytic activity of the 2D-WS_2 'per active site' was carried out using methodology reported by *Benck et al.*[52] and *Shin et al.*[66]. The true ink modification on the SPE working area surface will possess a finite roughness, but for the purpose of this calculation it is assumed that the 2D-WS_2 nanosheets surfaces are atomically flat.[52] The sulphur to sulphur bond distance was observed in literature to be 3.14Å,[67] leaving 4.269Å^2 to be the calculated value for the area per sulphur atom. This value can then be used to calculate the surface area occupied by each WS_2:

$$4.269\frac{\text{Å}^2}{S\ atom} \times \frac{2\ S\ atom}{1\ WS_2} = 8.538\frac{\text{Å}^2}{WS_2} \quad [1]$$

The number of surface sites for a flat standard i.e the derived area per WS_2 molecule as calculated above, is then used to determine the number of WS_2 molecules per cm^2 geometric area:

$$\frac{1\ WS_2}{8.538\text{Å}^2} \times \frac{10^{16}\text{Å}^2}{cm^2} = 1.171 \times 10^{15}\frac{WS_2}{cm^2} \quad [2]$$

The equation used to calculate the number of electrochemically accessible surface sites is then determined as follows:

$$\frac{\#\ Surface\ Sites\ (Catalyst)}{cm^2\ geometric\ area} = \frac{\#\ Surface\ sites\ (flat\ standard)}{cm^2\ geometric\ area} \times R_f \quad [3]$$

The roughness factor (R_f) of each WS_2 electrode must be determined in order to calculate the ToF 'per site': (See Roughness Factor calculation):

$$ToF\ per\ site = \frac{\#\ Total\ hydrogen\ turnovers\ per\ cm^2\ geometric\ area}{\#\ Surface\ sites\ (Catalyst)\ per\ cm^2\ geometric\ area} \quad [4]$$

The total number of hydrogen turnovers was calculated using the value of current density (mA cm^{-2}) at a potential of –0.51 V (*vs.* RHE), at a 25 mV s^{-1} scan rate. The answer models the 2D-WS_2-$SPE_{10\%}$ and utilises the following formula:

$$j\left(\frac{mA}{cm^2}\right)\left(\frac{1\,A}{1000mA}\right)\left(\frac{1C\,s^{-1}}{1\,A}\right)\left(\frac{1\,mol\,e^-}{96485.3\,C}\right)\left(\frac{1\,mol\,H_2}{2\,mol\,e^-}\right)\left(\frac{6.02214\times10^{23}}{1\,mol\,H_2}\right) = 2.187\times10^{15}\frac{H_2\,per\,S}{cm^2}\,per\,\frac{mA}{cm^2}$$

[5]

Using the value determined from formula 5, it is possible to calculate the ToF for each electrode in the following equation:

$$\left(2.187\times10^{15}\frac{H_{2\,per\,S}}{cm^2}\right)\left(10\frac{mA}{cm^2}\right)\left(\frac{1\,cm^2}{1.639\times10^{16}\,surface\,sites}\right) = 1.33\frac{H_2\,per\,S}{surface\,sites}$$

[6]

The current densities corresponded to –0.578, –0.701, –0.943 and −1.093 mA cm^{-2} for the 2D-WS_2-$SPE_{5\%}$, 2D-WS_2-$SPE_{10\%}$, 2D-WS_2-$SPE_{20\%}$, and 2D-WS_2-$SPE_{40\%}$, respectively. With these values the ToF values for each respective electrode was determined to be; 2.20, 1.33, 1.20 and $0.31\frac{H_{2\,per\,S}}{surface\,site}$.

Roughness factor calculation

Double layer capacitance is used to calculate the active surface area of the WS_2-SPEs via a method modified by *Rowley-Neale et al.*[38]. A non-faradaic window is determined in the potential range of 0.01 to +0.11 V, and cyclic voltammetry is performed at the following scan rates: 20, 40, 60, 80, 100 mV s^{-1}. It is assumed that within the potential range window of 0.01 to 0.11 V there are no faradaic processes, hence the anodic and cathodic current densities are responsible for the charging of the electrical double layer. (shown in Figure S2.) The potential difference between the anodic and cathodic current at 0.06 V against the relevant scan rate is shown in Figure S3, where the slope of each data set corresponds to a doubling of the double layer capacitance. The values for double layer capacitance were observed to be 7, 14, 21 and 95 μF cm^{-2} for the 2D-WS_2-$SPE_{5\%}$, 2D-WS_2-$SPE_{10\%}$, 2D-WS_2-$SPE_{20\%}$ and 2D-WS_2-$SPE_{40\%}$, respectively.

.

Figure S1. Raman maps displaying the surface of a (A) 2D-WS_2-$SPE_{5\%}$ (B) 2D-WS_2-$SPE_{10\%}$ (C) 2D-WS_2-$SPE_{20\%}$ and (D) 2D-WS_2-$SPE_{40\%}$, with the Raman intensity for each point representing the ratio between the sum of the characteristic WS_2 peak areas (351 cm^{-1}) and the single underlying area of the graphitic electrode peak (1580 cm^{-1}). The green maps represent the 2D-WS_2-SPEs and the black maps represent a bare/unmodified SPE surface. Both X and Y axes are in units of μm.

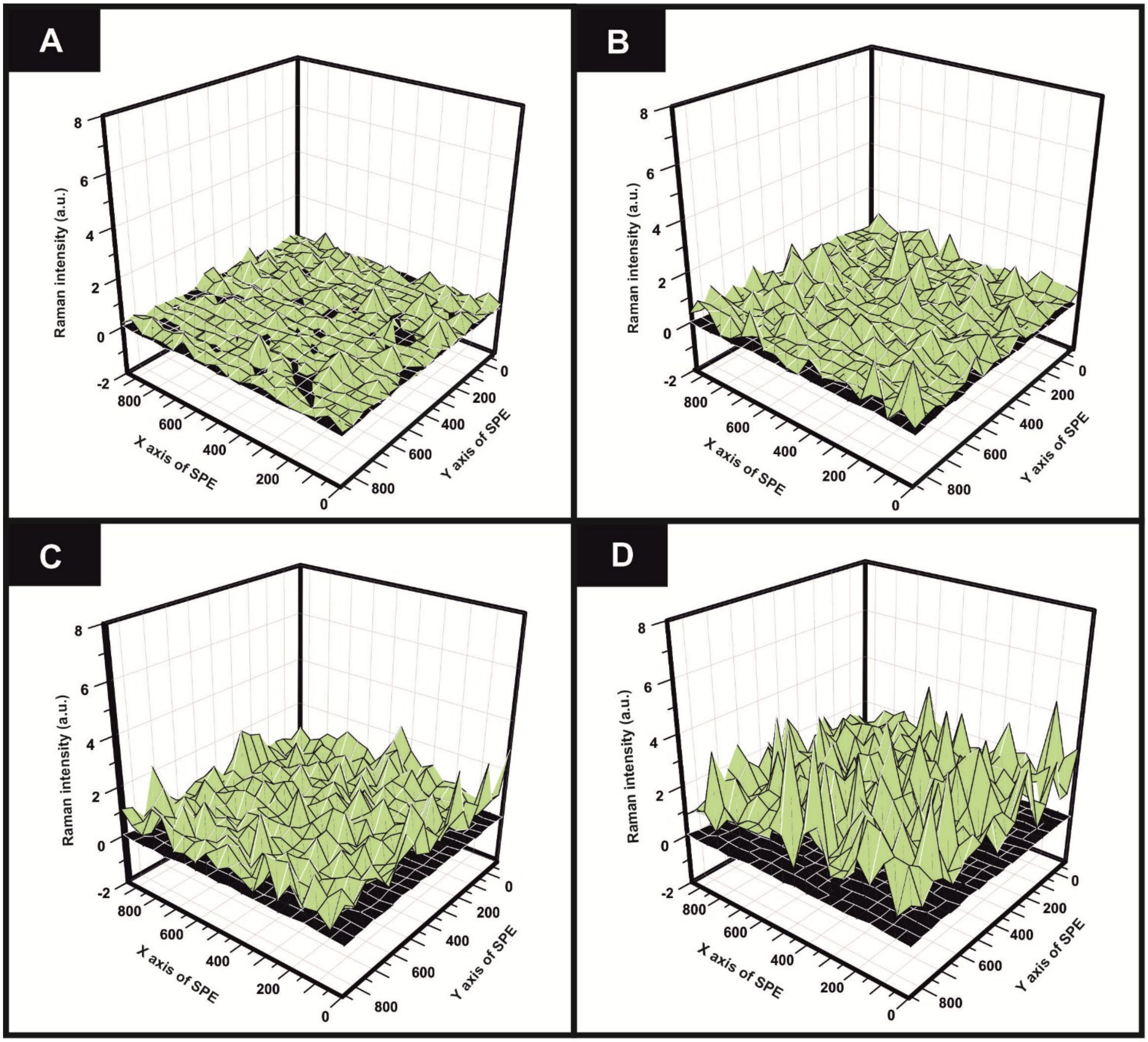

Figure S2. Cyclic voltammograms recorded in a 0.5 M H_2SO_4 electrolyte using the 2D-WS_2-$SPE_{5\%}$, 2D-WS_2-$SPE_{10\%}$, 2D-WS_2-$SPE_{20\%}$, and 2D-WS_2-$SPE_{40\%}$ in the scan rate range: 20-100 mV s^{-1}.

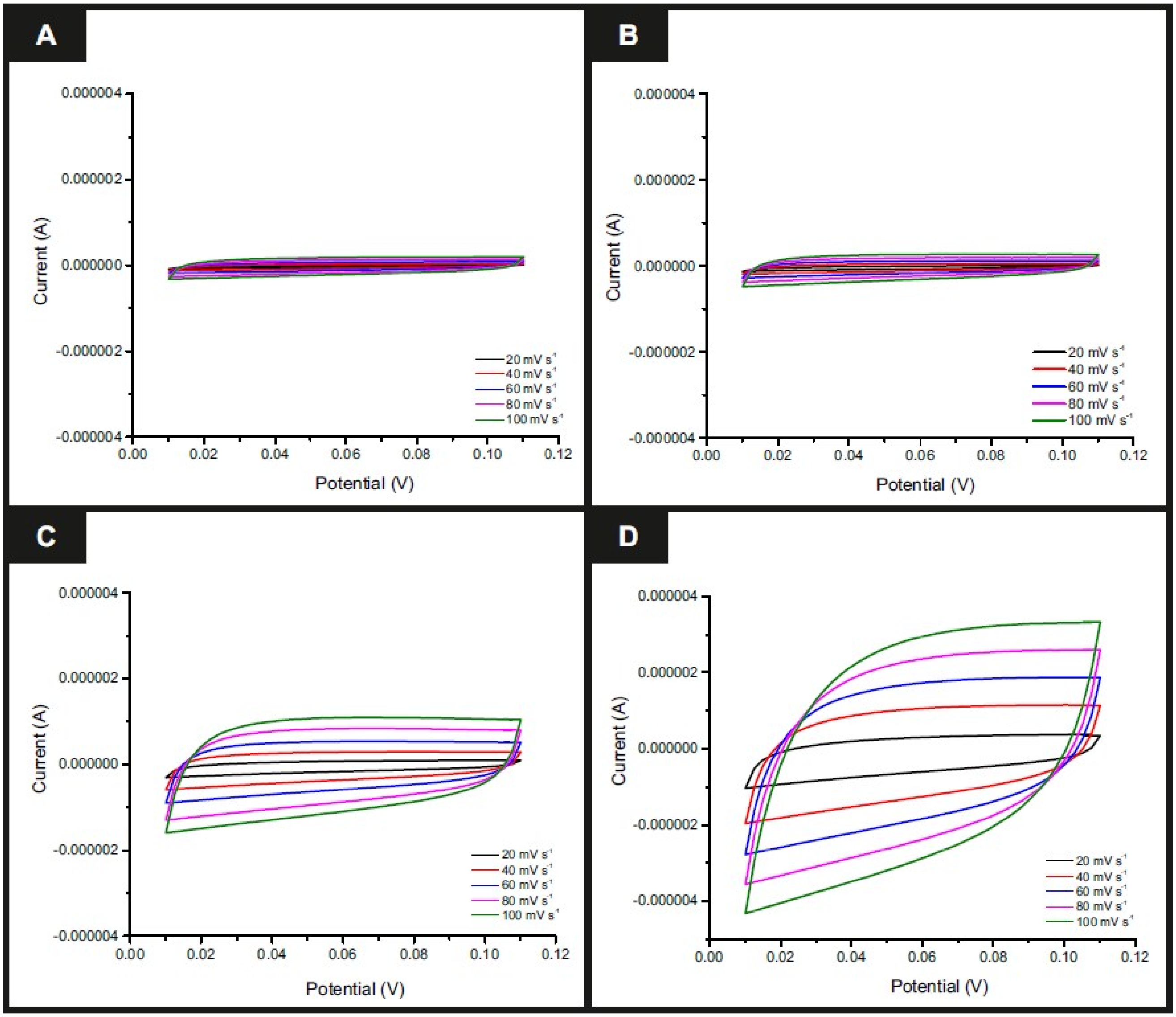

Figure S3. The difference in anodic and cathodic current density at a potential of +0.06 V against scan rate (mVs^{-1}) (*vs.* RHE). The slope of each data set indicates the value of double layer capacitance (C_{dl}: μF cm^{-2}).

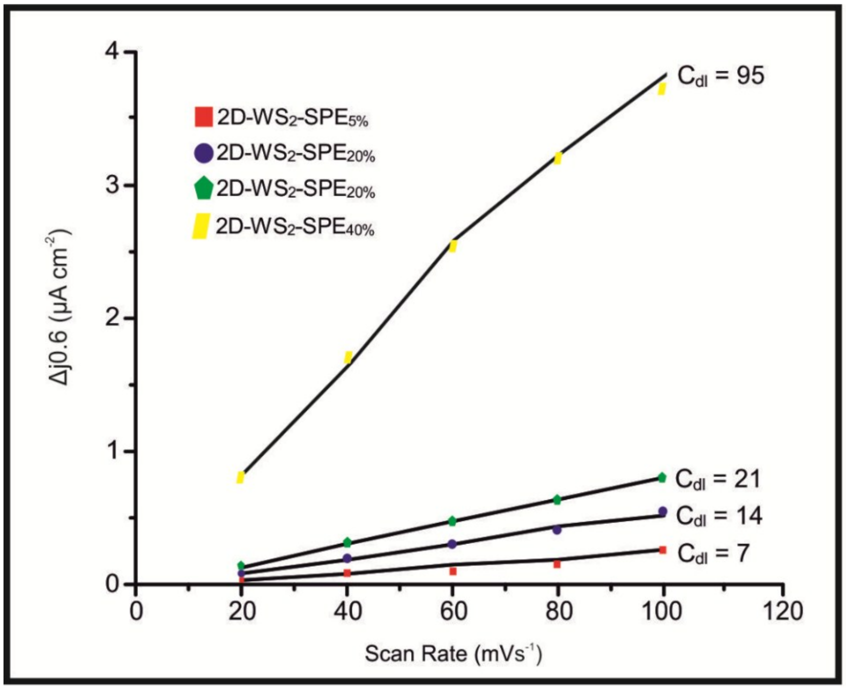

Supplement: RA-009-C9RA05342E-s001 [file RA-009-C9RA05342E-s001.pdf]
